# Evaluating link prediction by diffusion processes in dynamic networks

**DOI:** 10.1038/s41598-019-47271-9

**Published:** 2019-07-25

**Authors:** Didier A. Vega-Oliveros, Liang Zhao, Lilian Berton

**Affiliations:** 1 School of Informatics, Computing and Engineering, Indiana University, Bloomington, IN USA; 2 Department of Computing and Mathematics, University of São Paulo, Ribeirão Preto, SP Brazil; 3 Institute of Science and Technology, Federal University of São Paulo, São José dos Campos, SP Brazil

**Keywords:** Computer science, Information theory and computation, Statistical physics, Complex networks

## Abstract

Link prediction (LP) permits to infer missing or future connections in a network. The network organization defines how information spreads through the nodes. In turn, the spreading may induce changes in the connections and speed up the network evolution. Although many LP methods have been reported in the literature, as well some methodologies to evaluate them as a classification task or ranking problem, none have systematically investigated the effects on spreading and the structural network evolution. Here, we systematic analyze LP algorithms in a framework concerning: (1) different diffusion process – Epidemics, Information, and Rumor models; (2) which LP method most improve the spreading on the network by the addition of new links; (3) the structural properties of the LP-evolved networks. From extensive numerical simulations with representative existing LP methods on different datasets, we show that spreading improve in evolved scale-free networks with lower shortest-path and structural holes. We also find that properties like triangles, modularity, assortativity, or coreness may not increase the propagation. This work contributes as an overview of LP methods and network evolution and can be used as a practical guide of LP methods selection and evaluation in terms of computational cost, spreading capacity and network structure.

## Introduction

The emergence of social media has attracted considerable attention from researchers and companies. New platforms are continually emerging, e.g., Facebook and Flickr (2004), YouTube (2005), Twitter (2006), Sina Micro-blog (2009), among others. Given the relevance for different domains and areas, research topics such as Link Prediction (LP)^1–3^ and information diffusion^4–6^ have received substantial attention in complex and social networks area during the last years^2–4^. However, they are topics mostly studied in separated, even that their results are applied in similar domains, like viral marketing, political campaigns, and business process modeling.

The problem of recommending links has several applications, like suggesting missing and probable connections in noisy data^7^ or influential node identification^8^. In particular, the prediction of future links is helpful for the understanding of the network and communication evolution^9,10^. For example, in social media platforms, promising connections that not exist yet can promote engagement and interaction among users^9^, which also affects the network structure. In turn, the network structure impacts on the communication or the spread of information^4,5,11^.

LP methods estimate the new edges according to some connection strategies, like the distance and shortest paths among nodes, the triangles or triadic closure, the similarity with mutual neighbors, among others^1,2,7–9^. These structural factors are vital in interpreting networks evolution. For example, famous and influential users tend to gain more connections, creating traffic-based shortcuts and improving the efficiency of information spreading on the network^9^. Thus, analyzing the diffusion process can help to understand the impact of users interaction, e.g., how re-posting a message affects the spread of memes, videos, or fake news (rumors) on the networks. Users, in online social networks, not only make new friends but also seek and share information. When a user shares a message, his/her contacts can be influenced to re-post that information, driven by the homophily property that generates a diffusion process^6,9^.

On the other hand, diffusion processes on networks are essential for social science research, viral marketing applications, and epidemiology. Understanding how information spread has attracted much interest over the recent years, mainly because of the convenience of such predictions for effective marketing and political campaigns. Works in the area investigate which factors affect the reach of the diffusion^4^, how to effectively disseminate information^5,11^, which network properties promote a fast propagation^12^, and the influential capacity of nodes^13,14^. Although the previous studies helped to identify valuable insights into the diffusion processes, they disregard the effects of the dynamic evolution of connections.

Articles and methodology evaluations available on the literature focus on accurately estimate the classification performance over missing links^1–3^, but not the impact on the spreading capacity of the network. An initial study^9^ found clear evidence, in a meme dataset, that the spread of information affects the network evolution. Similar, in a micro-blogging platform, it was found that information diffusion affects the creation of new links^10^. The authors conclude, based on their data analysis, that including the diffusion process as a feature in the recommendation of new links performs better than only using topological properties. However, both works did not consider or evaluate LP strategies, neither a general method of evaluation or characterize the evolved networks. In the LP area, it was reported a measure based on the geometric mean of the AUC for evaluating the accuracy of LP methods^15^. They denominated ‘dynamic link prediction’ the problem of adding or removing recommended edges. Still, they did not consider any diffusion process, network evolution or structural characterization. In the line of the diffusion processes, two rewiring models were proposed to compare the effects on information spreading in scale-free and small-world networks^16^. However, the authors did not consider the addition of new edges, nor the effects on the structure of the networks. To the best of our knowledge, there have not been prior studies analyzing the role that LP methods, by the addition of new edges, have in the network and spreading capacity. Even more, a methodology that evaluates the LP methods concerning the information diffusion and structural evolution of the network. The evolutional behavior of adding links is common in complex networks^9,10,15^. For instance, online social networks are not static, growing over time through the addition of new edges, i.e., new friendships and collaborations are created continuously.

Here, we aim to investigate how the addition of new connections, guided by the LP methods, influence the information diffusion and structural evolution of the network. The addition of links can be associated with the speed up in the network evolution^9^, given that it impacts in the spreading capacity and topology of the network. We provide a comprehensive analysis of some representative local and global LP strategies, evaluating the structure of the evolved networks and, consequently, the effects on the diffusion process. Then, we categorize the LP methods based on their network structure and spreading, pointing the properties and strength of the methods.

**Figure 1:**
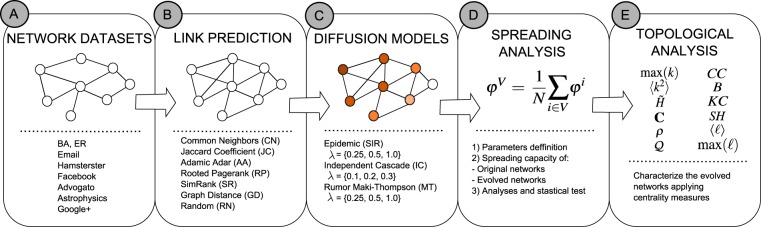
Workflow of the proposed method. (**A**) The employed datasets with two artificial–Barabási-Albert (BA)^21^ and Erdös-Rényi (ER)^20^ network models–and six real-world networks. (**B**) The evaluated LP methods: Common Neighbors (CN)^29^, Jaccard Coefficient (JC)^30^, Adamic Adar (AA)^31^, Rooted Pagerank (RP)^1,2^, SimRank (SR)^32^, Graph Distance (GD)^1,2^, and Random selection (RN); moreover, we produce LP-evolved networks with 1, 5, 10, and 20% of new edges. (**C**) The adopted diffusion models^4,17^ and the respective diffusion parameters: the epidemic Susceptible-Infected-Recovered (*SIR*), the information Independent Cascade (*IC*), and the rumor Maki-Thompson (*MT*) models. (**D**) The spreading capacity is the average size of outbreaks in terms of each node; the analyses and statistical test are performed in the evolved networks with respect to the original version. (**E**) The topological properties used for characterize the evolved networks: largest hub (max(*k*)), the complexity (*C*)^33^, the entropy ($\tilde{H}$)^34^ and second moment (〈*k*^2^〉) of degree distribution, the network assortativity (*ρ*)^22^, the modularity (${\mathscr{Q}}$)^22^, clustering coefficient (*CC*)^35^, the average of betweenness centrality (*B*)^33^, K-Core (*KC*)^4^, structural holes (*SH*)^36^, and shortest paths ($\langle \ell \rangle$), and the diameter (${\rm{\max }}(\ell )$) of the network.

We perform extensive simulations on two artificial network models and six real-world datasets (Fig. 1, box A), employ seven link prediction methods (Fig. 1, box B), and evaluate the spreading capacity of the network under three diffusion models (Fig. 1, box C). The list of LP methods is not exhaustive, due to the objective here is not to identify the best LP algorithm in the literature, but a method for evaluating the role that LP strategies have in the diffusion and structural evolution of the network. The main contributions of this work can be summarized as follow:

- To the best of our knowledge, this is the first study to evaluate the spreading capacity on evolving networks by LP methods. Moreover, our method is a new evaluation approach to the performance that LP methods have in the spreading and structural evolution of the network.
- We find by statistical tests that RP method enhances the spreading capacity with a significant difference, considering the evolved versions of the original networks. On the other hand, CN and AA are the worst methods for improving the spreading capacity.
- We characterize the evolved networks by the LP methods considering several centrality measures (Fig. 1 box E), showing that AA, CN, and GD generate new versions with higher degree complexity and triangles. On the other hand, RP has a minor impact on the complexity and assortativity of the evolved networks and a higher impact on the shortest paths and structural holes.
- We analyze the interplay between the network evolution and the diffusion process. The results indicate that evolved networks with lower shortest paths and structural holes have a higher spreading capacity.

## Results

We generate evolved versions of the original networks by adding a percentage of new predicted edges: 1, 5, 10, and 20%. The adopted LP methods produce the evolved versions (please, see Fig. 1, box B) for evaluating how the addition of edges improves the spreading capacity and affects the topological properties concerning the original network. Also, we consider as the baseline the random addition of links (RN).

For each node *i* ∈ *V*, with |*V*| = *N* the set of *N* nodes in the network, it is calculated the final fraction of informed individuals per node (*φ*^*i*^) according to some diffusion process. This *φ*^*i*^ means the information reach when *i* is the initial spreader, and it is an average over 1000 realizations. The *spreading* capacity of the network (*φ*^*V*^) is the average size of outbreaks for all nodes, i.e.,

1 $${\phi }^{V}=\frac{1}{N}\sum _{i\in V}{\phi }^{i},$$

which values are between [0, 1]. The spreading capacity quantifies the reach of the diffusion process on the network.

LP methods can serve as tools for enhancing and predicting the growth of social networks, e.g., satisfying user’s connectivity preferences and improving the spreading as the network evolves. For this reason, to understand the implications of how the evolution by adding new edges affect the network structure and spreading capacity, and how suitable are the LP methods to model this evolution, we divide the results into spreading and topological analyses.

### Spreading analysis

We consider the epidemic *SIR*, the rumor *MT*, and information Independent Cascade *IC* models^4,17^, according to Algorithm 1 in the Methods section. The transmission strength *λ* = *β*/*μ* is the ratio of the probability to inform over the probability to become stifler^12^. The *λ* can be seen as the maximum spreading potential of information. Without loss of generality, for *SIR* and *MT* simulations we adopt the *λ* = *β*/*μ* values [{0.25 = 0.2/0.8}, {0.5 = 0.4/0.8}, {1.0 = 0.4/0.4}]. For the *IC* simulations, we consider a global transmission probability between the nodes, with *β*_*ij*_ = *λ* = [0.1, 0.2, 0.3]. The spreading capacities are calculated as follows:

- Calculate the spreading capacity of the original networks according to each diffusion model.
- For each LP method, generate the evolved versions by including the respective percentage of new edges.
- Calculate the spreading capacity for each evolved network regarding each diffusion model.

#### Spreading results

**Figure 2:**
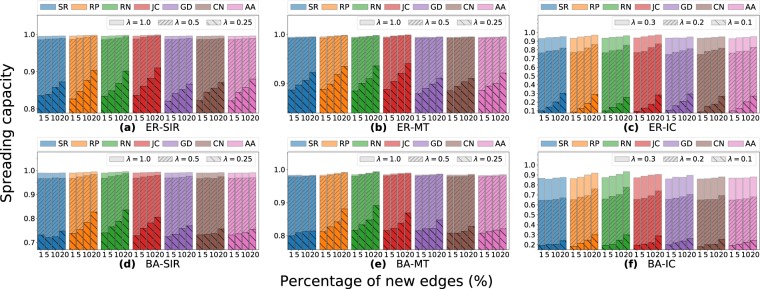
Effects on the network spreading capacity when adding new edges in the artificial networks. (**a**) to (**c**) correspond to the Erdös-Rényi networks, and (**d**) to (**f**) correspond for the Barabási-Albert networks. The spreading dynamics are the epidemic *SIR*, rumor *MT*, and information *IC* models. The edges increment are 1, 5, 10, and 20% of the original number of edges. The LP methods are the SimRank (SR), Rooted Pagerank (RP), Random Selection (RN), Common Neighbors (CN), Jaccard Coefficient (JC), Graph Distance (GD), and Adamic Adar (AA).

**Figure 3:**
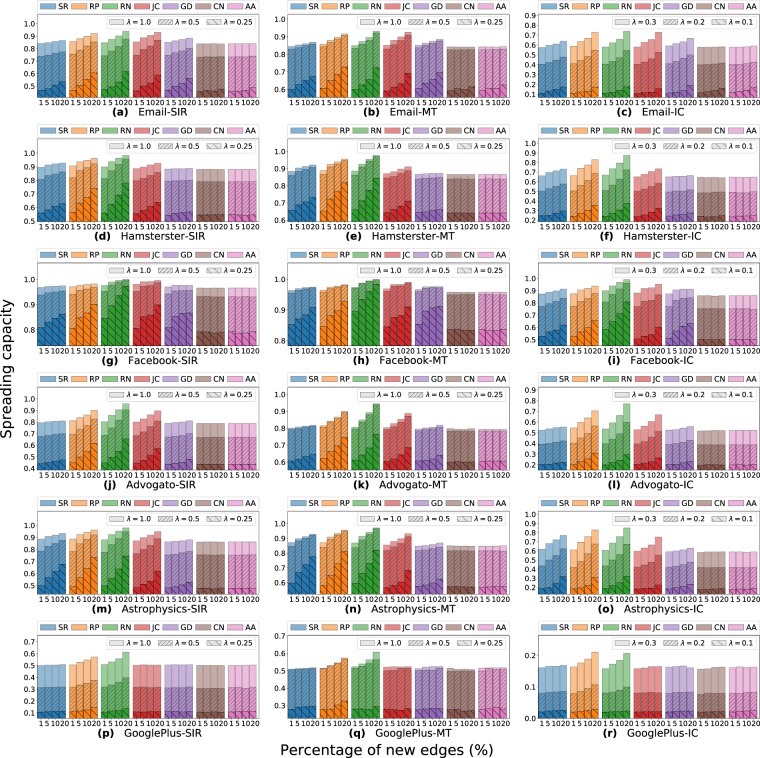
Effects on the spreading capacity when increasing the edges in the real-world networks in 1, 5, 10, and 20% with: the epidemic *SIR*, rumor *MT*, and information *IC* models; and the SimRank (SR), Rooted Pagerank (RP), Common Neighbors (CN), Jaccard Coefficient (JC), Graph Distance (GD), Adamic Adar (AA) and Random (RN).

The results for the artificial and real-world datasets are presented in Figs 2 and 3, respectively. In the ER networks, i.e., networks with more homogeneous degree distribution, the spreading capacities have a growing tendency when increasing the percentage of new edges, which is the expected behavior. On the other hand, in the BA networks, with more heterogeneous degree distribution, does not always happen the expected growing behavior, specifically for CN, and AA methods. The RP and JC methods always increase the spreading capacity in the presence of new edges. The increase in the spreading capacity is more evident for lower *λ* values (*λ* = 0.1 or 0.25 of Fig. 2). The before indicates that the diffusion dynamics for higher *λ* values are more likely to the saturation of spreaders in artificial networks, reaching very similar spreading results.

For real-world datasets (Fig. 3), in AA and CN methods the spreading capacity on the networks remains almost invariant. Different, the other methods show increasing results on the network spreading in the presence of new edges for most of the cases. The diffusion models show similar patterns of spreading among the LP methods, although the reached values are different. Even though SIR and *MT* use the same *λ* parameters, they obtain different spreading results. For example, in the *MT* simulations, with *λ* between 0.5 to 1.0, they have a much lower increment compared to *SIR* in the same cases. This result is due to the high number of informed nodes that cause the spreaders in *MT* turn inactive more quickly than SIR, then, the not significant growth. Opposite, the spreaders in the *MT* are longer activated than in the *SIR* case for the same low *λ* values, due to the smaller presence of informed individuals. Thereby, the simulations reach higher spreading capacity when *λ* = 0.25.

**Figure 4:**
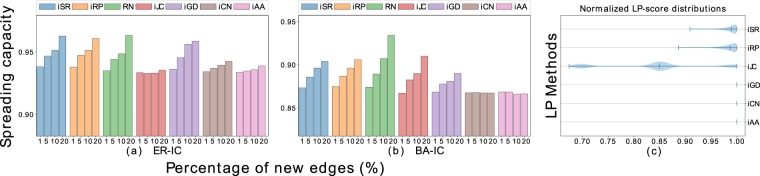
Effects on the spreading capacity according to the information *IC* model with *λ* = 0.3, when adding 1, 5, 10, and 20% of new links in the artificial networks. We consider the edges with the lowest, or inverse ranking, recommendation score from the methods: SimRank (iSR), Rooted Pagerank (iRP), Common Neighbors (iCN), Jaccard Coefficient (iJC), Graph Distance (iGD), and Adamic Adar (iAA). (**a**) Correspond to the Erdös-Rényi (ER) network; (**b**) correspond to the Barabási-Albert (BA) network; (**c**) shows the normalized distributions of the 20% worst LP-scores for the case of the BA network, where markers in between represent the median value of the distribution.

One may notice that the random addition of new links (RN) always improves the propagation of the network, outperforming, most of the times, the spreading capacity among all the discussed LP methods. Given that the LP methods recommend the edges according to some high-similarity criteria between nodes, we evaluate the case of adding the most unlikely new links for each method, i.e., the links with the lowest recommendation scores. In Fig. 4 are the results of the spreading capacities and the normalized distribution of the 1, 5, 10, and 20% lowest LP-scores for the artificial networks, concerning the *IC* model with *λ* = 0.3. We show this particular case due to, as pointed in Figs 2 and 3, the diffusion models and parameters show similar patterns of spreading capacity among the sub-figures. However, in the Supplementary information section are reported the results for the remaining real-world datasets.

We observe that both inverse rankings iAA and iCN little affect the spreading capacity, and the RN results are better than the inverse LP-scores ranking approach. Figure 4(c) shows the density distribution of the 20% inverse LP-scores for the BA network, in which the scores of iCN, iAA, and iGD are only one value. The iSR, iRP, and iJC also show low score dispersion, with most of the values centered on the median. This result indicates that the similarity-node criteria of the discussed LP methods, especially for iAA and iCN, neglect the scores for the worst edges recommendations, at least until the 20% of unlikely new links. However, iSR, iRP, and iJC present more notable dispersion scores in the inverse case. These results are similar to the remaining networks, which we also report in the Supplementary information section.

#### Statistical test

**Figure 5:**
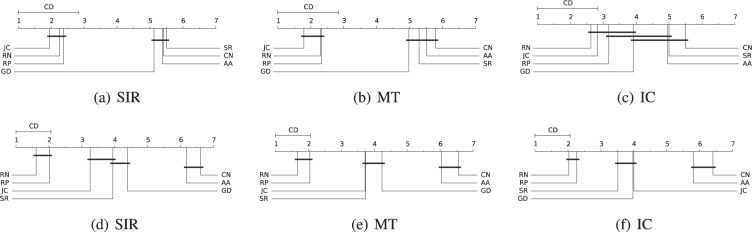
Statistical Nemenyi test for comparing the mean-ranking of the spreading capacity for the LP methods. The critical differences (CD) at 95 percentile are on top of the diagrams, where mean-ranking with significant differences are unconnected: (**a**) to (**c**) diagrams are for the artificial networks and (**d**) to (**f**) diagrams are for the real-world data sets. Methods in the lowest (best) positions are on the left side.

We perform statistical analyses over the spreading results of the LP methods to better understand the ranking and possible significant differences. We execute the Nemenyi post-hoc test^18^ grouping by diffusion models and separating between artificial and real-world networks, for all LP methods and evolved versions. The results for *SIR*, *MT*, and *IC* are shown in Fig. 5, where the figures in the top are for artificial and down for real-world networks. On the top of the diagrams is the critical difference (CD) and in the axis are plotted the average ranks of the LP methods, where the lowest (best) positions are on the left side. When a set of methods have no significant difference, they are connected by a black line in the diagram.

For the artificial networks, according to the Friedman test^19^ using the F-statistics with 6 and 138 degrees of freedom and at 95 percentile, the critical value is 2.16 and the null-hypothesis that all methods behave similarly should be rejected. Running the Nemenyi post-hoc test to detect differences among the LP methods, the CD for comparing the average ranking of two different LP methods at 95 percentile is 1.84. For the real-world networks, the critical value of the F-statistics with 6 and 426 degrees of freedom at 95 percentile is 2.12 and, the null hypothesis that all methods behave similarly should be rejected. In the Nemenyi statistics, the CD for comparing the mean-ranking of two different methods at 95 percentile is 1.06. Mean-rankings differences above the CD value are significant.

According to the Nemenyi results for the artificial networks, Fig. 5(a–c), the JC and RP methods are the best-ranked for improving the spreading capacity of the evolved versions, with JC been the best ranked in *SIR* and *MT* diffusion models. Besides, the AA, CN, and SR are the worst positioned with statistical ranking differences compared to JC and RP. In the case of real-world networks, Fig. 5(d–f), RN and RP are always the best ranked with no significant difference between them, but to other methods. The JC, SR, and GD are in the ranking group of second best spreading capacity, which perform significantly better to the third and last group, the AA and CN methods.

We observe that the order of the methods in the ranking changes depending on the diffusion model. However, in general, the first places ranked methods are the same. The intervals of significant difference connecting the methods and some positions change in the ranking with respect to the diffusion models. For instance, the JC and SR are both in the third place of the ranking for the *MT* model, but JC and GD are both in the fourth place for the *IC* model. Finally, what is a consensus in the statistical tests is that CN and AA are the latest classified, with the worse ranking positions.

We summarize the effects of adding new edges on the networks according to LP methods concerning the spreading capacity results, as follow:

- The LP methods that seem no affect the spreading capacity are AA and CN. Both are local methods that consider the common neighbors between pairs of nodes. In this case, the addition of new edges little or nothing improves the spreading capacity of the network.
- The LP methods that most of the times have an increasing pattern in the spreading capacity are SR, GD, and JC. The first two are global methods, and the last is a local similarity index. They show increases or holds in the spreading capacity of networks when adding new edges.
- The LP method that always increases the spreading capacity is RP, which is a global index. In this case, the higher the percentage of new edges, the greater the spreading capacity. Also, the random addition of connections always performed well in the diffusion models.

Here was performed an exhaustive analysis producing four evolved versions for each network of box A from Fig. 1, and for each of the seven LP methods; the simulations concerning the three diffusion models and the three *λ* parameter combinations lead to a total of 2016 spreading capacity simulations. However, we show that the results are similar in terms of the diffusion model, *λ* parameters, and percentage of new edges. Thus and in practical terms, the evaluation can be led by selecting a lower *λ* parameter, one of the diffusion model, and a particular percentage of new edges.

We also observed that the spreading capacity is affected in different ways, depending on the LP method. It is known that the structure of the network plays an essential role in the dynamical diffusion processes^4^, where some properties, like the node localization^12^, communities or network assortativity^5,11^, make the spreading more viral or slow by a fire-wall effect^4,12^. This way, the LP methods can be biased to produce evolved versions tending to a specific network organization. In the next Section, we analyze the structural properties of the original, and the LP generated networks.

### Topological analysis

We analyze the central-point increase of the structural properties comparing the new version with the original network, i.e., for a particular topological property, we have the *increase* (*x*_*e*_ − *x*_*o*_)/*x*_*o*_, where *x*_*e*_ is the measurement of the evolved network and *x*_*o*_ of the original. We have the following considerations:

- The number of nodes is constant and the percentage of new edges is the same in all the cases. Thereby, we can obtain constant values of average degree in each edge increment among the LP methods.
- We consider the set of topological properties described in the box E of Fig. 1. For more details of the network measures and selection, please see the Supplementary information section.
- For each original and evolved network, we measure its structure according to the set of topological properties. Then, we analyze the central-point increase on the LP-evolved networks.

Given that we have the same set of points in the x-axis (percentages) and all the cases start in (0, 0) (the original network), we calculate the linear regression between the central-points obtaining the slope of the curves. After that, we measure the angle from the slope in radians into the range (−1, 1), in the way:

2 $${\rm{\Theta }}=\arctan ({\rm{slope}})/(\pi /\mathrm{2).}$$

Thereby, we obtain a representation of the slopes in the same range measuring the *tendency* of how “not flat” are the curves, with a positive or negative growing inclination.

#### Topological results

**Figure 6:**
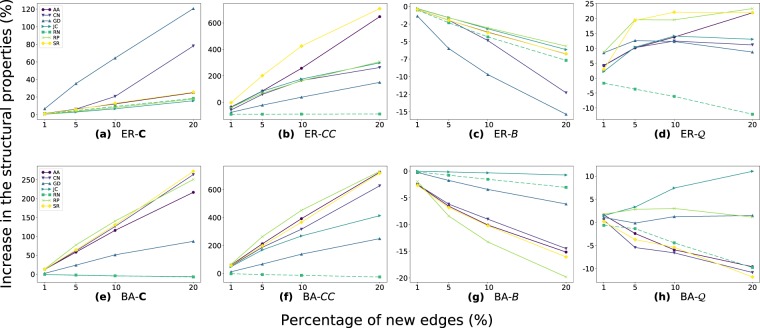
Evolution of the topological properties for artificial networks. (**a**) to (**d**) are for Erdös-Rényi (ER) and (**e**) to (**h**) are for Barabási-Albert (BA) artificial models. The networks evolve by favoring new connections between nodes according to the LP methods. The percentages of new edges (*x*-axes) are 1, 5, 10, and 20%. Each figure shows the increase/decrease pattern for a specific measure, which are: network complexity (**C**) ((**a**,**e**)), clustering coefficient (*CC*) ((**b**,**f**)), average betweenness centrality (*B*) ((**c**,**g**)), and network modularity (${\mathscr{Q}}$) ((**d**,**h**)). The increase/decrease values are with respect to the original network property.

Figure 6 shows the structural evolution for the artificial networks, with the complexity (Fig. 6(a,e)), clustering coefficient (Fig. 6(b,f)), average betweenness centrality (Fig. 6(c,g)), and modularity of the best community division (Fig. 6(d,h)). Excepting the modularity evolution, the methods present similar patterns of increasing/decreasing behavior, but with different intensity. When adding new edges, the methods affect in different ways the modularity of the artificial networks, and the patterns are not always the same between the ER and BA networks. For example, SR has a positive increase in the ER, but negative in BA. In general, *C* and *CC* increase and *B* decreases in the network evolution according to the LP methods. RN and JC methods little increase the network complexity when adding new edges. The growth in *C* implies a more extensive degree heterogeneity, i.e., an increase of the second moment of the degree distribution that leads to a strong presence of hubs on the network. However, we can see that the random addition of edges little increases the *C* property. The before is given the low probability to randomly select a hub on the network to assign it a new edge. RN and GD are the methods that least impact in the *CC* of the evolved versions. Besides, we notice the substantial increase of the methods in the *CC*. The high increase is due to the low proportion of triangles in the artificial networks, as described in Table 2 of the Methods section.

**Figure 7:**
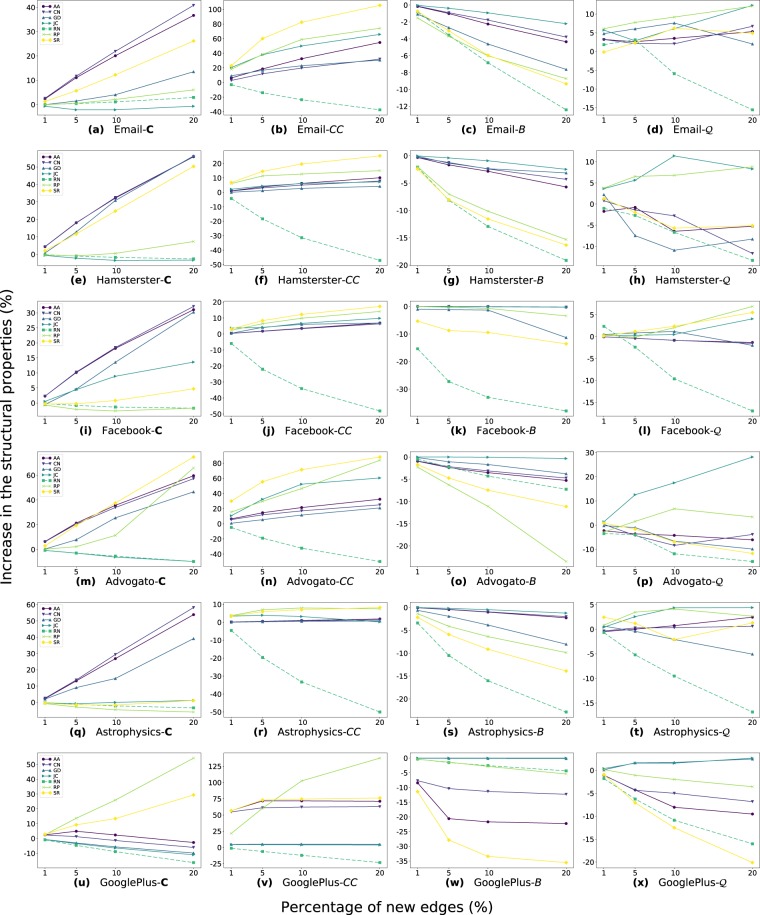
Evolution of the topological properties for the real-world network datasets: the networks evolve by favoring new connections between nodes according to the LP methods. The *x*-axes are the percentage of new edges. Each figure is a data set that shows the increase/decrease pattern for a specific measure according to the LP method and the central-point to the original network.

In the real-world datasets (Fig. 7), RN is the only one that always has decreasing patterns in the structural properties. AA and CN are those that more increase the network complexity (The *C* column in Fig. 7), opposite to JC with a little increase in most of the networks. Regarding the proportion of triangles, except for RN, all methods have a positive tendency of increasing the *CC* when adding the recommended edges, with SR and RP the ones that increased the most. Besides, a drop pattern of *B* happens when adding the predicted edges. The before implies that the number of shortest paths distributes more homogeneously in the network, decreasing the importance of nodes with high betweenness centrality. JC is one of the least affect *B* of the evolved networks. On the other hand, SR is one of the most decreases the *B*, and most increase the *CC*. We could suppose that higher the number of triangles, lower the average betweenness centrality of the network. However, this is not true, and the counterexample is JC, where it considerable increases *CC* on the Advogato and Email networks (Fig. 7(b,n)) but the impact on the *B* was minimal.

#### Topological characterization

**Figure 8:**
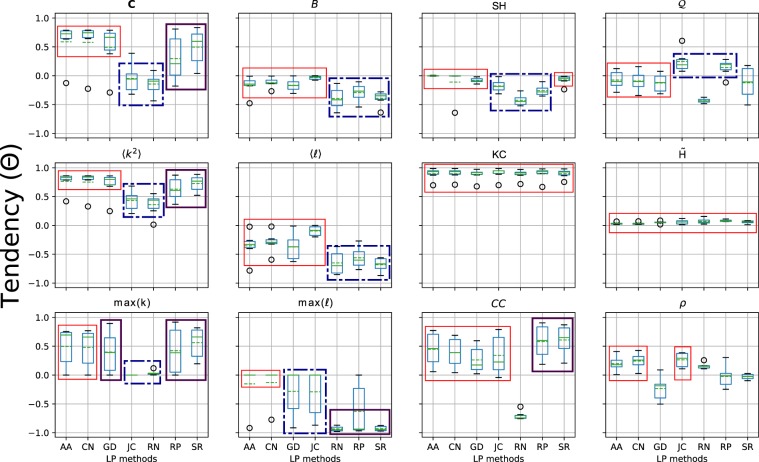
Distribution of the increase tendencies for the LP methods grouped by topological measures. In the BoxPlots, the dotted lines are the arithmetic mean, and the continuous lines are the median. In each figure, methods with a similar pattern of increase tendency are encapsulated in the same frame.

The results in Fig. 7 indicate that the LP methods affect in different ways the network structure by the addition of the recommended edges. For a better understanding of the topological characteristics that each LP methods favor in the evolved networks, we calculate the slope tendency (Eq. 2) for all the set of topological properties and real-world datasets. Then, with the box plots, we show the distribution of tendencies, grouping by network property (Fig. 8). Inside the boxes, the median is the continuous line and the dotted line is the arithmetic mean.

First, we observe that the *KC* and $\tilde{H}$ of the evolved networks do not change. We identify groups of methods that tend to produce networks with similar structural tendencies. The AN, CN, and GD tend to highly increase the complexity of the network and 〈*k*^2^〉. They are the group that less impact the modularity and the structural holes, together with SR. Also, this group together with JC have similar increasing patterns in the shortest paths and betweenness centrality, with low decreasing tendency; in *CC* and *ρ*, they have a low increasing tendency, but GD has an opposite behavior to the group in the assortativity.

In the evolution of network connectivity (first column of fig. in 8), JC and RN produce networks with the lowest or negative growing tendency in the 〈*k*^2^〉 and *C*, and no changes in the maximum degree of the network. On the other hand, RP and SR have an intermediate impact on the connectivity of the network compared to the other methods. For the shortest path evolution, (second column of fig. in 8), RN, RP, and SR have the lowest median and mean values. This result indicates that in the evolved networks, the nodes are more close to each other, with lower diameter, shortest path, and betweenness centrality average. Similar, JC, RN, and RP are the methods with the lower structural holes mean and median values (*SH* in Fig. 8).

In the *CC* measure, RP and SR highly increase, and RN most decreases *CC*. The decreasing effect by RN is due to the random recommendation of edges that not follows any connectivity pattern of the nodes. Thus, the new edges impact negatively in the average proportion of triangles of the nodes. In the modularity ${\mathscr{Q}}$, JC and RP have certain increase tendency; and RN and SR present negative tendency. In the assortativity, RN has a low increase tendency; RP and SR, on average, little affect the degree correlation, but RP has higher dispersion than SR. These results demonstrate that each LP method generates networks with different topological characteristics.

### The interplay between network evolution and spreading

We summarize in Table 1 the results obtained in the spreading and topological analysis, together with the time complexity of each LP method. The columns of table are: **C**, as the measure that resumes the effects on network connectivity by the LP methods; *CC*, the measurement that shows the impact in the proportion of triangles among the nodes; *B*, representing the influence in the shortest-path-oriented measures on the network; ${\mathscr{Q}}$, showing the evolution in the community division; *SH*, which shows the average of all possible structural holes; and the *ρ*, which brings how the LP methods influence in the degree-degree correlation.

**Table 1:** Summary of LP methods and effects in the structure and spreading capacity of the network.

*Method*	Computational cost	C	*CC*	*B*	${\mathscr{Q}}$	*SH*	*ρ*	Spreading capacity
CN	*O*(*N*〈*k*〉^2^)	+++	++	−	−	°	+	Ultra low increase
AA	*O*(*N*〈*k*〉^2^)	+++	++	−	−	°	+	Ultra low increase
GD	*O*(*N*^2^)	++	+	−	−	−	−−	Low increase
JC	*O*(*N*〈*k*〉^2^)	−	**++**	°	**+**	−	+	Medium increase
SR	*O*(*N*^3^)	++	+++	−−	−	−	°	Medium increase
**RP**	**O**(**N**^**3**^)	**+**	**+++**	**−−**	**+**	**−**	°	**High increase**
**RN**	**O**(**1**)	**−**	**−−−**	**−−**	**−−**	**−−**	**−−**	**High increase**

+++ High increase tendency. −−− High decrease tendency.

++ Medium increase tendency. ° No pattern. −− Medium decrease tendency.

+ Low increase tendency. − Low decrease tendency.

In contrast to what is expected, including more edges on the networks will not necessarily improve the spreading capacity. All the methods included the same amounts of new links, but CN and AA reached ultra low increases in the spreading capacity. Opposite, RP and RN are the both that more increase the spreading of the evolved networks. Seeking a better understanding between the structure evolution and the diffusion dynamic, we describe the structural properties that increment the spreading capacity in terms of the LP methods:

- *More homogeneously distributed networks*: RN, JC, and RP are the methods with lower central tendencies in *C*. They are the methods that less enlarge the 〈*k*^2^〉. The maximum degree (hub) of the network is not affected by JC and RN.
- *The lower distance among the nodes*: RN, RP, and SR present the lower central tendencies in *B*, $\langle \ell \rangle$, and ${\rm{\max }}(\ell )$. The before indicates that lowering the average distance between the nodes can prone the information diffusion in the network. However, the increase in the number of triadic closure properties did not show an increment pattern with the spreading capacity of the network.
- *The lower average of structural holes*: The LP methods with lower central tendencies in the *SH* are in a similar order than the ranking of spreading capacity enhancers (Fig. 5(d,f)). Thus, decreasing the *SH* of the evolved network can improve the reach of the spreading process.

Besides, some structural properties do not increase/decrease the spreading. The measures *CC*, ${\mathscr{Q}}$, and *ρ* do not show a clear pattern concerning the improvement of the spreading capacity of the networks. For instance, increasing the number of triangles did not improve the spreading results for the AA. Moreover, RP and RN have opposite tendency behavior in *CC*. The measures that are not affected at all for the LP methods, neither characterize any pattern for improving the spreading results are the *KC* and $\tilde{H}$. Additionally, concerning the computational cost of the methods, the local measures tend not to affect the spreading capacity, while global methods, such as RP, SR, and GD, always increase the spreading capacity. A particular case is JC, which takes into consideration local information for predicting the links. It obtained a remarkable performance in the artificial networks, but in the real datasets its performances are behind SR. However, JC is less time-consuming.

## Discussion

More and more LP methods are being developed and reported in the literature. Each of them has its particularities and may lead to different prediction results. Therefore, how to choose a suitable LP method to achieve a specific global network structure and dynamics turns out to be a critical issue in real applications. This work presents an endeavor in this direction, analyzing how the network evolution by the addition of new edges affects the spread of information. For this purpose, we consider the most representative LP methods for increment the edges (please, see Fig. 1). Note that our aim is not necessarily to identify the most accurate LP method, but rather to establish a mechanism and a set of general recommendations for future considerations.

Using the proposed framework in two artificial and six real-world networks, and with three diffusion approaches–epidemic *SIR*, rumor *MT*, and information *IC* models–the experimental results reveal that methods, like RP, RN, JC, and SR always lead to an increase in the spreading capacity. RP is one of the most increases the spreading on the network. Contrary with expected, the inclusion of more edges may not improve the spreading capacity of the evolved versions. For instance, CN and AA little impact on the spreading results, with performances worst than the random addition of edges and all other methods. Concerning the computational cost, RP obtained better spreading results than JC, but the more suitable option would be JC given that it is less time-consuming.

In terms of structural properties, LP methods that retain or decrease the network complexity obtain better spreading results. The increase in the number of triangles, changes in the modularity or assortativity regarding the original network do not show any pattern concerning the spreading capacity. This result is also in contrast to what is expected. The measures that are not affected at all for the LP methods are the K-Core and the entropy of degree distribution. The results also indicate that evolved networks with lower shortest paths and structural holes averages present a higher spreading capacity.

The proposed method is suitable as a methodology for comparing the diffusion potential that LP methods have when predicting future edges. This evaluation is relevant in network sciences, given that the recommendation of links directly affects the propagation process due to the changes in the network structure. In turn, the diffusion process may provoke changes in the connections and speed up the network evolution. Therefore, the LP methods impact in different ways the structure and spreading capacity of the network. Most studies and evaluation proposals in LP methods ignore this point, and here, we make a contribution in this direction. Furthermore, the proposed methodology can be applied in other works as an alternative for evaluating current and new LP methods.

Finally, we contribute to the understanding of how to spread information more widely on the network when adding new connections, and the interplay between the evolution of the structure and the diffusion process. As future works, novel and more accurate LP methods that improve the spreading capacity of the networks opens a new path of studies and possibilities for higher performance in evolutionary networks.

## Methods

We introduce some basic definitions about LP methods and the diffusion models employed for measuring the spreading capacity of the networks.

### Problem definition

Given a network *G* = (*V*, *E*), where *V* is the set of |*V*| = *N* nodes and *E* is the set of links connecting pairs of nodes, the LP method calculates the likelihood of new edges for each node pair (*i*, *j*) ∉ *E* at future time. The methods recommend the links based on higher score or predicted likelihood. We consider the original network in the last observed discrete state. The performed task seeks to predict new edges in a future state of the network, i.e., the evolved network contains an increment of edges concerning its previous state. We perform the network evolution considering fixed fractions of new predicted edges by some LP method, as shown in Fig. 1 box B. Then, we analyze the spreading capacity and structural properties of the evolved versions, verifying statistical differences and characterizing the methods.

### Dataset

We adopt the Erdös-Rényi (ER)^20^ and Barabási-Albert (BA)^21^ models as artificial networks. These two models are representative regarding networks characteristics. The ER networks present Poisson degree distribution, while the BA networks have power-law degree distribution. For the same input of nodes and edges, both models generate similar artificial networks concerning average degree, modular structure, and triangles proportion. However, artificial networks have an absence of important properties present in real-world networks, like the clustering and the community structure^22^.

We adopt six real-world network datasets: Email^23^, Hamsterster^24^, Facebook^24^, Advogato^25^, Astrophysics^26^, and GooglePlus^27^. Email represents a social network of information exchanged by emails between members of the *Rovira i Virgili* University, Tarragona. The Hamsterster, an undirected and unweighted network based on the user-user friend and family social network website data from hamsterster.com. Facebook contains friendship data of Facebook users from New Orleans regional network. The Advogato, nodes are users of an online community platform for developers of free software, and the edges represent trust relationships. GooglePlus, an user-user social network that denotes when a user has the other in his circles. Also, Astrophysics, a collaborative network between scientists on previous studies of astrophysics reported in arXiv. We considered the main component of datasets as undirected and unweighted for the simulations.

The topological characteristics of these networks are summarized in Table 2, with the measures: number of nodes (*N*), average degree (〈*k*〉), largest degree (max(*k*_*i*_)), network complexity (**C**), second moment of degree distribution (〈*k*^2^〉), average betweenness centrality of the network (*B*), clustering coefficient (*CC*), and modularity (${\mathscr{Q}}$).

**Table 2:** Topological properties of the adopted networks.

Network	*N*	〈*k*〉	max(*k*_*i*_)	C	〈*k*^2^〉	*B*	CC	${\mathscr{Q}}$
BA	10000	11.9	777	48.4	581.5	11572,7	0.015	0.25
ER	10000	12.0	27	13.0	156.1	14823,6	0.001	0.24
Email	1133	9.60	71	18.6	179.8	1475.2	0.22	0.49
Hamsterster	2000	16.1	273	43.7	704.7	2587.7	0.54	0.46
Facebook	4039	43.7	1045	106.6	4656.1	5436.1	0.60	0.78
Advogato	5054	15.6	807	82.8	1290.4	5747.7	0.25	0.34
Astrophysics	14845	16.1	360	45.4	732.3	28190,4	0.66	0.63
GooglePlus	23613	3.30	2761	377.2	1251.6	35804.7	0.17	0.74

### Link prediction methods

LP methods recommend potential links in the network using the topological information represented by the adjacent matrix *A*^*N*×*N*^. The result is a link similarity score matrix *S*^*N*×*N*^, where *s*_*ij*_ indicates the probability of the existence of links between nodes *i* and *j*. The higher the *s*_*ij*_, the higher the likelihood of the link between the nodes. When the similarity *s*_*ij*_ is based only on network structure, it is called structural similarity^2^. Authors usually classified the methods on local or global information techniques^2,3^.

Here, we employ three of the more representative local and global LP methods (please, see box B of Fig. 1), as classical approaches recommended by some reviews and surveys^1,2^: from local measures, we select CN, JC, and AA; from global measures, we select the RP, SR, and GD. These are also the most well-known LP methods in the area, and they represent main strategies across many other methods, i.e., triangle, paths, or neighborhood optimization. Moreover, exploring mechanisms underlying network evolution, we can analyze the influence of main strategies to increase the links in the networks and how these strategies influence the network topology and the information diffusion. We briefly describe the adopted methods in the Supplementary information.

### Diffusion process

The common epidemic spreading approach is the Susceptible-Infected-Recovered (*SIR*)^4,17^ model. A pathogen spreads from infected users to susceptible users, as information is dispersed from communicators to recipients in a similar fashion. In the *SIR* model, the recovered individuals are those infected that obtained immunity to the pathogen. In information diffusion, it means those that spontaneously do not spread the information anymore^4^. In this context, the propagation of rumors or information can be approached as a psychological contagion where an idea “contaminates” the mind of other people^4^. The psychological contagion reflects the several factors in which an individual is predisposed to adopt and disseminate a message, like social reinforcement, homophily phenomenon, curiosity, etc.^6,11,17^.

In the case of the Maki-Thompson (*MT*) rumor model^4,12,28^, in the ignorant or inactive state (*S*) remain those who are unaware of the rumor, in the spreader (*I*) or active state are those who disseminate the message, and the recovery or stifler (*R*) state are those who know the rumor but lose the interest in spreading it. Rumor models are a variant of the *SIR* model in the sense that a rumor disperses intentionally and the recovery process does not occur spontaneously, but the transition between states is a consequence of contact interaction^4^.

Moreover, we have the Independent Cascade (*IC*) model for information spreading, which is an inhomogeneous *SIR* approach^4^. This model assumes that the spreading process is an informational cascade of activation^17^. Such cascades consist of the successive activation of nodes that know the information and subsequently contact their neighbors according to an activation rule. In the *IC* model, whenever a node becomes active (*I*), it tries to activate its inactive neighbors (*S*) according to some probability *β*_*ij*_ and then stops. An active node cannot be deactivated; however, it does not participate anymore in the remaining steps of the process (as the recovery state for epidemic spreading).

We summarize in general terms the dynamical rules of the three previous diffusion models as follow:

3 $${I}_{i}+{S}_{j}\mathop{\to }\limits^{{\beta }_{ij}}{I}_{i}+{I}_{j},$$

where *i* and *j* are neighbors and the operator “+” means the contact action between them. In the diffusion models, whenever an active spreader *I*_*i*_ contacts an inactive neighbor *S*_*j*_, the latter will become active with a fixed probability *β*_*ij*_. This probability for activation/infection can be global, for all the nodes (*β*_*ij*_ = *β*), or specific for each pair (*i*, *j*)^12,17^.

Otherwise, in Eq. 4 for the *SIR* and *IC* models^4,17^, the active spreader *I*_*i*_ stops propagating the information according to a spontaneous probability *μ*. In particular, for the *IC* model the active node stops immediately after contacting all its neighbors^17^, i.e., with *μ* = 1.

4 $${I}_{i}\mathop{\to }\limits^{\mu }{R}_{i}$$

On the other hand, in Eq. 5 for the *MT* rumor model, when an active spreader *I*_*i*_ makes contact with a neighbor *j* that knows about the rumor, i.e., *j* is a spreader (*I*_*j*_) or a stifler (*R*_*j*_), the *I*_*i*_ node will turn into a stifler with probability *μ*. This stopping behavior means that the information is assumed too much known (by contacting spreaders) or without novelty (by contacting stifler) by the spreader^12,28^.

5 $$\{\begin{array}{lll}{I}_{i}+{R}_{j} & \mathop{\to }\limits^{\mu } & {R}_{i}+{R}_{j},\\ {I}_{i}+{I}_{j} & \mathop{\to }\limits^{\mu } & {R}_{i}+{I}_{j}\end{array}$$

Let us consider a constant population of *N* nodes in all time steps. Each node can be only in one state, i.e., node *i* is a spreader at time *t I*_*i*_(*t*) = 1 *if i* ∈ *I* in t, otherwise *I*_*i*_(*t*) = 0, and the states are discrete with *S*_*i*_(*t*) + *I*_*i*_(*t*) + *R*_*i*_(*i*) = 1. Therefore, we describe the macroscopic state of the system over time with the spreaders (*ϕ*(*t*)) and stifler (*φ*(*t*)) fractions,

6 $$\begin{array}{rcl}\varphi (t) & = & \frac{1}{N}\sum _{i\in V}{I}_{i}(t),\\ \phi (t) & = & \frac{1}{N}\sum _{i\in V}{R}_{i}(t),\end{array}$$

where the fraction of ignorant subjects (*ψ*(*t*)) always fulfill *ψ*(*t*) = 1 − (*ϕ*(*t*) + *φ*(*t*)). Moreover, the end of the propagation occurs when achieved the absorbing state of the system, i.e., when no more spreaders exist in the dynamic with *ϕ* = 0 for *t* → ∞. We assume a synchronous dynamical process, in which infection and recovering do not occur during the same step.

We present in Algorithm 1 a general approach employed for simulating the *SIR*, *MT*, and *IC* models. The algorithm receives as inputs the network *G*, the set of initial states of the nodes *S*(0), *I*(0) and *R*(0), the propagation probabilities, and the type of model to be simulated. The sets of node states can be addressed as structured lists at a specific time. At each time step, each spreader node tries to activate or infect its neighbors (in lines (7–9) of Algorithm 1). After that, the spreader evaluates if will become recovery or inactive in the diffusion process. The simulations run until the end of the propagation process is reached, when *ϕ*(*t*) = 0. The Infect-Node procedure is the general rule of activation/infection in the diffusion models (Eq. 3), in which the probability of propagation *β* can be a general or specific value. The Recover-Node procedure handles the inactivation rule for the spreader according to the particular diffusion model, as explained in Eqs 4 and 5.

For illustrative purposes, steps (18–19) and (38–39) in Algorithm 1 are the similar procedure of updating the state of the nodes, which can be generalized in a separate updating function. Moreover, the algorithm can be easily optimized by only counting the number of infected/recovery individuals disregarding the time evolution arrays. The computational cost of the algorithm is $O(TN\langle k\rangle )$ (similar to^6^), where *T* represents the number of required steps for convergence. However, *T* tends to be low, which means the computational cost can be approximated to $O(N\langle k\rangle )$.

**Algorithm 1:**
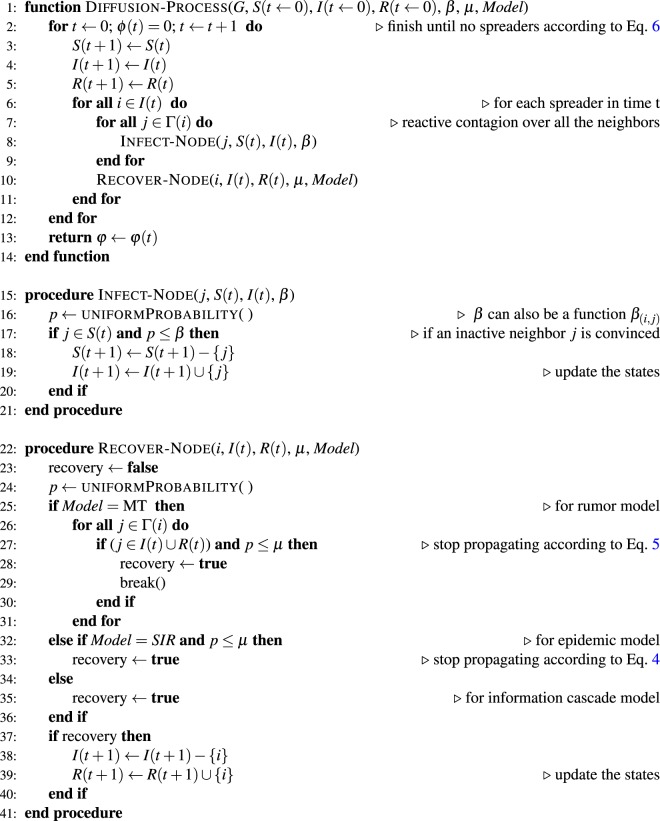
General diffusion process algorithm.

## Supplementary information

Supplementary information

## Data Availability

The datasets analyzed during the current study are available at http://konect.uni-koblenz.de.
