# Supplementary material for: Evaluating link prediction by diffusion processes in dynamic networks
Source: Sci Rep. 2019 Jul 25;9:10833. doi: 10.1038/s41598-019-47271-9 (PMC6658485; doi:10.1038/s41598-019-47271-9)
Supplement: Supplementary file 1 — Supplementary information [file 41598_2019_47271_MOESM1_ESM.pdf]

Evaluating link prediction by diffusion processes in dynamic networks

Supplementary information

Didier A. Vega-Oliveros[1,2*], Liang Zhao[2], and Lilian Berton[3]

[1]School of Informatics, Computing and Engineering - Indiana University, Bloomington, IN - USA

[2]Department of Computing and Mathematics - University of São Paulo, Ribeirão Preto, SP - Brazil

[3]Institute of Science and Technology - Federal University of São Paulo, São José dos Campos, SP - Brazil

[*]Corresponding author: davo@icmc.usp.br

July 8, 2019

S.1 Supplementary material of the spreading results

We evaluate the impact of adding new edges in the spreading capacity of the networks, by considering the most unlikely links according to the LP methods, i.e., the lowest recommendation scores.

In Figure 1 are the results of the spreading capacities and in Figure 2 the normalized distribution of the 20% lowest LP-scores for all the datasets. In these simulations, we consider only the *IC* model with $\lambda = 0.3$, due to the patterns of the spreading capacities of the LP methods are similar among diffusion models and propagation parameters. This point is better discussed in the main paper.

We observe that iAA and iCN little affect the spreading capacity of the evolved networks, opposite to iRP, iSR, iGD, and iJC, in that order (Figure 1). However, the results

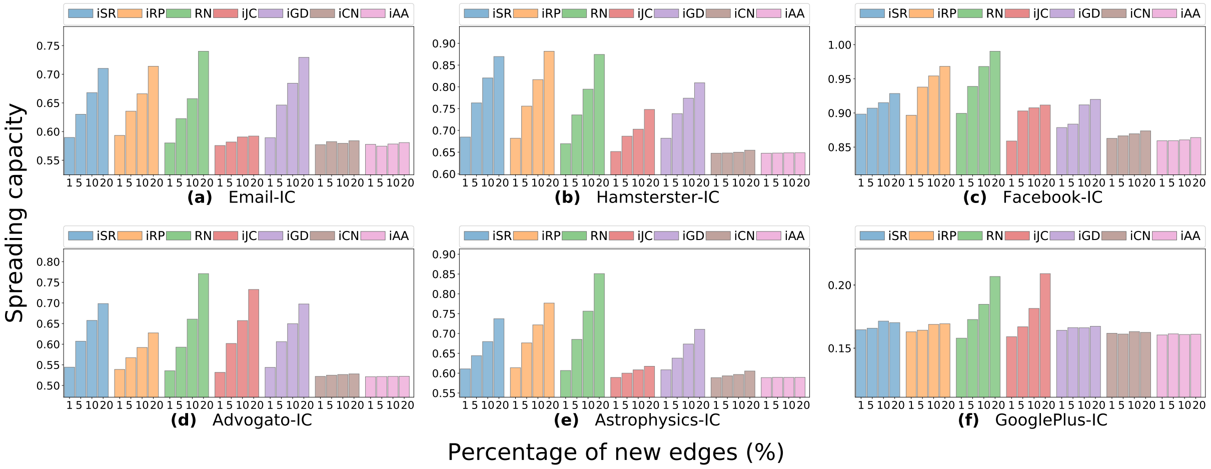

Figure 1: Effects on the spreading capacity according to the information IC model with $\lambda = 0.3$, when adding $1, 5, 10$, and 20% of new links in the real-world networks: (a) Email [5]; (b) Hamsterster [8], (c) Facebook [8]; (d) Advogato [11]; (e) Astrophysics [14], and (f) GooglePlus [12]. We consider the edges with the lowest recommendation score from the methods, as inverse: SimRank (iSR), Rooted Pagerank (iRP), Common Neighbors (iCN), Jaccard Coefficient (iJC), Graph Distance (iGD), and Adamic Adar (iAA). RN is the random addition of new links.

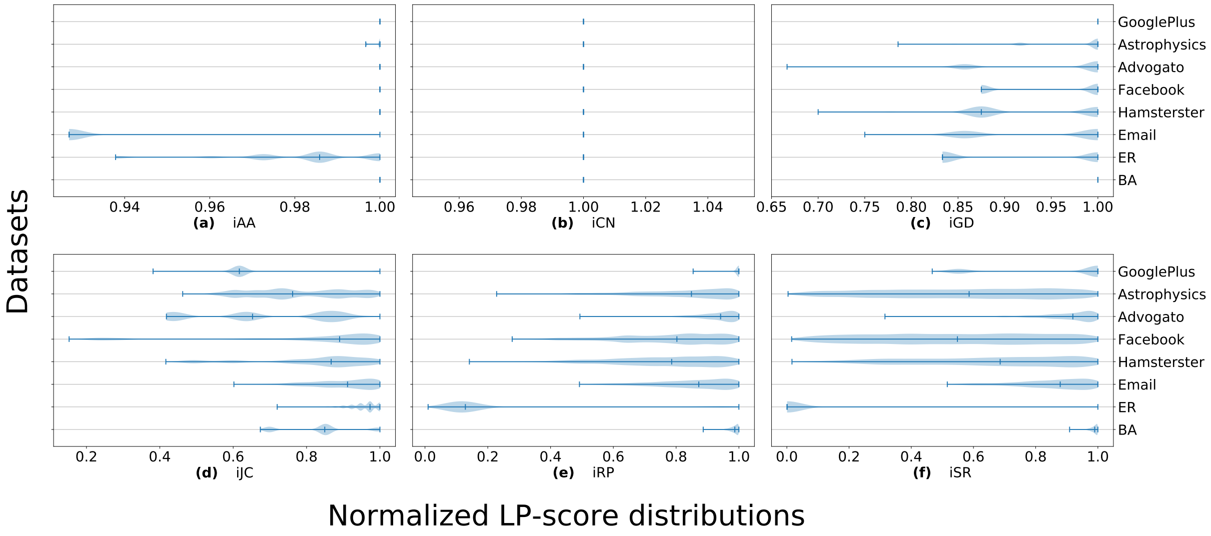

Figure 2: Normalized dispersion distributions of the 20% lower LP-scores across the considered network datasets, where markers in between represent the median point of the distribution.

of RN are better than the inverse LP-scores approach. In terms of the LP-score distribution, (Figure 2) iCN and iAA present the lowest dispersion of score, with all the 20% of lowest scores equal to some value in iCN, and in iAA most of the values equal to the median. iSR, iRP, and iJC present more notable dispersion in the 20% of lowest values.

S.2 Network sciences concepts

Several measures have been proposed for network characterization [16]. It is important to consider a set of features of the network, like the heterogeneity and dispersion of the degree distribution, the number of triangles, the proportion of shortest paths, and the community or modular structure, among others.

In a undirected networks $G = (V, E)$, where V is the set of nodes and E is the set of links, *the degree connectivity* of node i, called as k_i, is the number of links or connections incident on i. In addition, we denote $\Gamma(i)$ as the set of neighbors of node i, in which $|\Gamma(i)| = k_i$. Hubs are the nodes that have a very high degree in the network. The *degree distribution* of an undirected network $P(k)$ is the probability of randomly select a node with degree k, or for finite networks, as the fraction of nodes with degree equal to k [16]. Social networks exhibit heavy-tailed degree distribution following a power law [13] in the form $P(k) \sim k^{-\gamma}$, in which most of the individuals have a low degree, and few of them have a very high degree (hubs). When the exponent of the power-law takes values $2 \leq \gamma \leq 3$, it is called as a scale-free network [16]. Most of the real-world social networks follow a scale-free degree distribution [15].

We can calculate the moments of the degree distribution according to

$$\langle k^n \rangle = \sum_{k=0}^{\infty} k^n p(k), \tag{1}$$

in which *the first and the second moment of the degree distribution* are the average degree (for $n = 1$) $\langle k \rangle = 2M/N$, and the dispersion (for $n = 2$) $\langle k^2 \rangle$, respectively. The average degree provides information about the density of the network. On the other hand, the second moment of the degree distribution provides a central variance, which diverges in the presence of very heterogeneous networks, capturing large connectivity fluctuation associated with heavily-tailed distributions.

We characterize the level of heterogeneity through the heuristic *Network Complexity* measure, according to [2]:

$$\mathbf{C} = \frac{\langle k^2 \rangle}{\langle k \rangle}, \text{ with } \mathbf{C} \geq 0, \tag{2}$$

which is the rate among the second and the first moment of the degree distribution. The normalization of the variance denotes the fluctuations in the network complexity. **C** is close to zero when the network follows a more regular degree distribution. Opposite, for

scale-free networks, the second moment of degree distribution diverges ($\langle k^2 \rangle \to \infty$) in the infinite network size ($N \to \infty$). However, in the case of Poisson distributed networks, $\langle k^2 \rangle \sim \langle k \rangle^2$ [2].

Other options to measure the level of disorder or similarity of connection patterns between nodes are the entropy and degree-degree correlation. The normalized version of the Shannon entropy [21] ($\tilde{H}$) is useful for measuring *the entropy of degree distribution*, i.e.,

$$\tilde{H} = -\frac{\sum_{k=0}^{\infty} P(k) \log(P(k))}{\log(N)} , \tag{3}$$

with $0 \leq \tilde{H} \leq 1$. The more the disparity in degree connections, the higher the entropy, with maximum value occurring with a uniform $P(k)$. The lowest possible entropy happens when all nodes have the same degree. The other network property is *the degree-degree correlation* (or *assortativity*), in which nodes with similar degree tend to be connected. The level of assortativity (ρ) can be quantified by the degree Pearson correlation coefficient [13]. According to this measure, a network will be classified as assortative, or positively correlated, with $\rho > 0$; disassortative, low-degree nodes tend to connect with strongly connected pairs, with $\rho < 0$; or non-assortative, with no connection pattern and $\rho \approx 0$.

Concerning the structural analysis, the network can be decomposed into shells or cores [18]. *The K-Core* ($\mathrm{K} \in \mathbb{N}_{>0}$ or core of order K) is the maximum subset of nodes that have at least degree $k_i \geq \mathrm{K}$ and the K-Core is the highest-order core they belong to [18]. Nodes with the highest coreness are the most central. However, Nodes located at the periphery of the network have lower K-Core centrality, even hubs located in this region. We denote KC as the average of K-Core centrality among the nodes.

The presence of triangles is a common property found in real-world networks [14]. In topology terms, it is the number of cycles of order three on the network [2, 14]. These triangles have shown to be a relevant feature for complex systems, and more important to social networks [15, 24]. They are related to the similarity or homophily connection between individuals [15, 19] and how cohesive are the social circles [6]. Here, we characterized the triangles by the *Clustering Coefficient*, which is the average of triangles proportion for each node [22]

$$CC = \frac{1}{N} \sum_{i \in V} \frac{2t_i}{k_i(k_i - 1)} , \tag{4}$$

where k_i is the degree of node i and t_i is the number of triangles centered on i. In the case of $k_i = \{0, 1\}$, it is assumed the value of the fraction equal to zero. The maximum value happens when all the neighbors of i are interconnected.

Some nodes work as a bridge between clusters or other nodes, and when removed, a structural hole will occur. Nodes that act as structural holes are spanners among communities or groups of nodes without direct connections. These individuals are essential to the connectivity of local regions. *The structural holes centrality* [3] considers the ego network of each node, ignoring connections no related to it. Nodes with higher degree centrality have low values for structural-holes centrality. The low centrality value is because hubs present more extensive and densely interconnected ego networks, and this factor diminishes the presence of isolated holes. We denote SH as the average of the structural holes centralities of all the nodes.

Concerning the network distances, *the shortest path* or geodesic path ℓ_{ij} is the shortest distance between two nodes. In a global scale, we can compute the average shortest paths ($\langle \ell \rangle$) by measuring how close are the nodes to each other. Besides, we have *the diameter of the network* ($\max(\ell)$), which is the longest of all shortest paths and represent the linear size of a network.

The proportion of shortest paths in which a specific node appears is related to the capacity of information transmission of that node [16]. Thus, *the betweenness centrality* of a node j is the ratio of the number of shortest paths between all pairs of nodes $(i,\, l)$ that contain j [4], where i, j and l are different. The betweenness centrality has revealed to be important for understanding many dynamical and complex systems [16]. For example, in social sciences, it was found that intrepid individuals were less likely to cluster, but more likely to feature high betweenness centrality by connecting different communities [6].

We characterize the overall betweenness of the network (B) by performing the average among the nodes, mathematically:

$$B = \frac{1}{|V|} \sum_{i \neq j,\, i \neq l}^{V} \frac{\sigma_{i,l}(j)}{\sigma_{i,l}}, \text{ with } i, j, l \in V, \tag{5}$$

where $\sigma_{i.l}$ is the total number of different shortest paths between i and l, and $\sigma_{i,l}(j)$ is the number of times j appears in those paths.

Last, the community structure is another critical feature of the networks, which impacts in the performances of the propagation process [20, 23]. Communities are sets of

densely interconnected nodes and sparsely connected with the rest of the network [13]. One of the most relevant measure for evaluating the community structure of the networks is the $\mathcal{Q}$ *modularity* [13]. It compares the density of intra-community and inter-community edges relative to a non-correlated random network of similar size. Moreover, this measure is employed by several techniques to identify communities in networks systems, especially in divisive and agglomerative approaches [2, 5, 13].

S.3 Description of the adopted LP methods

Jaccard Coefficient (JC): It is used to account the probability for randomly select a node that is a neighbor of i and j from the set of all neighbors between nodes i and j [17]. Therefore, the higher the probability, the higher the similarity between the nodes.

$$s_{i,j}^{JC} = \frac{|\Gamma(i) \cap \Gamma(j)|}{|\Gamma(i) \cup \Gamma(j)|} \tag{6}$$

Common Neighbors (CN): it refers to the size of the set of all common neighbors of both i and j [9].

$$s_{i,j}^{CN} = |\Gamma(i) \cap \Gamma(j)| \tag{7}$$

Adamic Adar (AA): it refines the simple counting of common neighbors by weighting rarer neighbors more heavily [1].

$$s_{i,j}^{AA} = \sum_{z \in \Gamma(i) \cap \Gamma(j)} \frac{1}{\log(k_z)} \tag{8}$$

Rooted Pagerank (RP): it is a ranking method that inherently scales according to node distance [25]. Define by $s_{i,j}$, the probability that a random walker starting from node i locates at node j in the steady state results in [10]:

$$\begin{aligned} s_{i,j}^{RP} &= s_{i,j} + s_{j,i} \ , \\ \vec{s}_i &= (1-\alpha)(I - \alpha P^T)^{-1} \vec{e}_i \end{aligned} \tag{9}$$

where P is the transition matrix.

SimRank (SR): its a random walk on the collaboration graph: the expected value

of ι^l, where l is a random variable giving the time at which random walks started from i and j first meet [7].

$$s^{SR}_{i,j} = \iota . \frac{\displaystyle\sum_{z \in \Gamma(i)} \sum_{z' \in \Gamma(j)} s^{SR}_{z,z'}}{k_i . k_j} \;, \tag{10}$$

where $s^{SR}_{i,i} = 1$ and $\iota = [0, 1]$ is the decay factor [10].

Graph Distance (GD): it follows the small world properties and seeks to recommend pairs of nodes that are closely connected:

$$s^{GD}_{i,j} = -\ell_{i,j} \tag{11}$$

This way, the negative sign ensure the increasing order of the shortest path distances $s^{GD}_{i,j}$ for closer pairs $(i,\ j)$.

References

[1] L. A. Adamic and E. Adar. Friends and neighbors on the web. *Social Networks*, 25(3):211–230, 2003.

[2] A. Barrat, M. Barthélemy, and A. Vespignani. *Dynamical processes on complex networks*. Cambridge University Press, 2008.

[3] R. Burt. *Structural holes: The social structure of competition*. Harvard University Press, Cambridge, MA, 1992.

[4] L. C. Freeman. Centrality in Social Networks: Conceptual Clarification. *Social Networks*, 1:215–239, 1979.

[5] R. Guimera, L. Danon, A. Diaz-Guilera, F. Giralt, and A. Arenas. Self-similar community structure in a network of human interactions. *Physical Review E*, 68:2003, 2003.

[6] A. Ilany and E. Akçay. Personality and Social Networks: A Generative Model Approach. *Integrative and Comparative Biology*, 56(6):1197–1205, dec 2016.

[7] G. Jeh and J. Widom. Simrank: A measure of structural-context similarity. In *Proceedings of the Eighth ACM SIGKDD International Conference on Knowledge Discovery and Data Mining*, KDD '02, pages 538–543, New York, NY, USA, 2002. ACM.

[8] J. Kunegis. The koblenz network collection – KONECT, jan 2019.

[9] F. Lorrain and H. White. Structural equivalence of individuals in social networks. *Journal of Mathematical Sociology*, 1:49–80, 1971.

[10] L. Lü and T. Zhou. Link prediction in complex networks: A survey. *Physica A*, 390(6):1150 – 1170, 2011.

[11] P. Massa, M. Salvetti, and D. Tomasoni. Bowling alone and trust decline in social network sites. In *2009 Eighth IEEE International Conference on Dependable, Autonomic and Secure Computing*, pages 658–663, Dec 2009.

[12] J. J. McAuley and J. Leskovec. Learning to discover social circles in ego networks. In P. L. Bartlett, F. C. N. Pereira, C. J. C. Burges, L. Bottou, and K. Q. Weinberger, editors, *Advances in Neural Information Processing Systems 25: 26th Annual Conference on Neural Information Processing Systems 2012. Proceedings of a meeting held December 3-6, 2012, Lake Tahoe, Nevada, United States.*, pages 548–556. 2012.

[13] M. Newman. *Networks: An Introduction.* Oxford University Press, Inc., New York, NY, USA, 2010.

[14] M. E. J. Newman. The structure of scientific collaboration networks. *Proceedings of the National Academy of Sciences USA*, 98:404–409, 2001.

[15] M. E. J. Newman and J. Park. Why social networks are different from other types of networks. *Phys. Rev. E*, 68(3):036122, sep 2003.

[16] R. Pastor-Satorras, C. Castellano, P. Van Mieghem, and A. Vespignani. Epidemic processes in complex networks. *Reviews of Modern Physics*, 87(3):925–979, aug 2015.

[17] G. Salton and M. J. McGill. *Introduction to Modern Information Retrieval.* 1983.

[18] S. Seidman. Network structure and minimum degree. *Social Networks*, 5(3), 1983.

[19] D. A. Vega-Oliveros, L. Berton, F. Vazquez, and F. A. Rodrigues. The impact of social curiosity on information spreading on networks. In *Proceedings of the 2017 IEEE/ACM International Conference on Advances in Social Networks Analysis and Mining*, ASONAM '17, pages 459–466, 2017.

[20] D. A. Vega-Oliveros, L. da F Costa, and F. A. Rodrigues. Influence maximization on correlated networks through community identification. *arXiv*, page 1705.00630, may 2017.

[21] B. Wang, H. Tang, C. Guo, and Z. Xiu. Entropy optimization of scale-free networks' robustness to random failures. *Physica A: Statistical Mechanics and its Applications*, 363(2):591–596, 2006.

[22] D. Watts and S. Strogatz. Collective dynamics of 'small-world' networks. *Nature*, 393(6684):440–442, 1998.

[23] L. Weng, F. Menczer, and Y.-Y. Ahn. Virality prediction and community structure in social networks. *Scientific reports*, 3(1):2522, jan 2013.

[24] L. Weng, J. Ratkiewicz, N. Perra, B. Gonçalves, C. Castillo, F. Bonchi, R. Schifanella, F. Menczer, and A. Flammini. The role of information diffusion in the evolution of social networks. In *Proceedings of the 19th ACM SIGKDD international conference on Knowledge discovery and data mining - KDD '13*, page 356, 2013.

[25] Y. Yang, R. N. Lichtenwalter, and N. V. Chawla. Evaluating link prediction methods. *Knowledge and Information Systems*, 45(3):751–782, dec 2015.
